# Computational Modeling for Antiarrhythmic Drugs for Atrial Fibrillation According to Genotype

**DOI:** 10.3389/fphys.2021.650449

**Published:** 2021-05-13

**Authors:** Inseok Hwang, Ze Jin, Je-Wook Park, Oh-Seok Kwon, Byounghyun Lim, Myunghee Hong, Min Kim, Hee-Tae Yu, Tae-Hoon Kim, Jae-Sun Uhm, Boyoung Joung, Moon-Hyoung Lee, Hui-Nam Pak

**Affiliations:** Yonsei University Health System, Seoul, South Korea

**Keywords:** atrial fibrillation, modeling, antiarrhythmic drugs, *PITX2*, gene

## Abstract

**Background:** The efficacy of antiarrhythmic drugs (AAD) can vary in patients with atrial fibrillation (AF), and the *PITX2* gene affects the responsiveness of AADs. We explored the virtual AAD (V-AAD) responses between wild-type and *PITX2*^+/−^-deficient AF conditions by realistic *in silico* AF modeling.

**Methods:** We tested the V-AADs in AF modeling integrated with patients' 3D-computed tomography and 3D-electroanatomical mapping, acquired in 25 patients (68% male, 59.8 ± 9.8 years old, 32.0% paroxysmal type). The ion currents for the *PITX2*^+/−^ deficiency and each AAD (amiodarone, sotalol, dronedarone, flecainide, and propafenone) were defined based on previous publications.

**Results:** We compared the wild-type and *PITX2*^+/−^ deficiency in terms of the action potential duration (APD_90_), conduction velocity (CV), maximal slope of restitution (Smax), and wave-dynamic parameters, such as the dominant frequency (DF), phase singularities (PS), and AF termination rates according to the V-AADs. The *PITX2*^+/−^-deficient model exhibited a shorter APD_90_ (*p* < 0.001), a lower Smax (*p* < 0.001), mean DF (*p* = 0.012), PS number (*p* < 0.001), and a longer AF cycle length (AFCL, *p* = 0.011). Five V-AADs changed the electrophysiology in a dose-dependent manner. AAD-induced AFCL lengthening (*p* < 0.001) and reductions in the CV (*p* = 0.033), peak DF (*p* < 0.001), and PS number (*p* < 0.001) were more significant in *PITX2*^+/−^-deficient than wild-type AF. *PITX2*^+/−^-deficient AF was easier to terminate with class IC AADs than the wild-type AF (*p* = 0.018).

**Conclusions:** The computational modeling-guided AAD test was feasible for evaluating the efficacy of multiple AADs in patients with AF. AF wave-dynamic and electrophysiological characteristics are different among the *PITX2*-deficient and the wild-type genotype models.

## Introduction

Atrial fibrillation (AF) is found in 1.6% of the overall population, and the prevalence is steadily increasing with the aging population (Kim et al., 2018). Because AF is a progressive chronic disease, the more AF progresses to the persistent form, the more difficult it is to control the rhythm (Calkins et al., 2017). On the other hand, active rhythm control of AF, including catheter ablation, helps prevent AF complications such as ischemic strokes, dementia, heart failure, and renal failure (Friberg et al., 2016; Marrouche et al., 2018; Jin et al., 2019; Noseworthy et al., 2019; Park et al., 2019). Nevertheless, using drugs for AF rhythm control is not easy because of the low efficacy and safety of antiarrhythmic drugs (AAD) (Singh et al., 2005). AF has been proven to be a heritable disease (Lubitz et al., 2010). Although it is still disputable, the genetic characteristics might play an essential role in AAD responsiveness and AF recurrence in *de novo* AF or after electrical cardioversion (Parvez et al., 2012). Previous studies reported that a *PITX2*^+/−^*-*deficient condition modulated atrial resting membrane potentials and increased both AF susceptibility and the efficacy of AADs, especially class IC drugs (Syeda et al., 2016; Bai et al., 2021) and that dronedarone restored the action potential of myocytes affected by *hERG* mutations to that of wild-type (WT) myocytes (Loewe et al., 2014). Amiodarone has also been found to decrease APD heterogeneity and to affect AF termination (Varela et al., 2016). However, it is not clinically possible to predict the efficacy of toxic AADs prior to dosing. With the development of high-speed parallel computing hardware system (Boyle et al., 2019), AF simulation modeling has become more efficient for clinical applications. In particular, we previously reported that AF catheter ablation results can be predicted and improved by using computational modeling before the procedure (Kim et al., 2019). In this study, we created the realistic atrial model by reflecting personalized electroanatomy and modulating the specific ion currents for AADs. We then compared the effects of AADs based on wild-type and *PITX2*^+/−^-deficient AF models. The purpose of this study was to evaluate whether computational modeling of AAD study was a useful method for studying AF susceptibility and dose-dependent responses of virtual AADs.

## Materials and Methods

### Ethical Approval

The study protocol adhered to the Declaration of Helsinki and approved by the Institutional Review Board of the Severance Cardiovascular Hospital, Yonsei University Health System. All participants provided written consent for the researchers to access their genetic data, CT images, and clinical mapping data. Patients that participated in the study were included in the Yonsei AF Ablation Cohort Database (ClinicalTrials.gov Identifier: NCT02138695).

### Realistic AF Modeling

**Figure 1:**
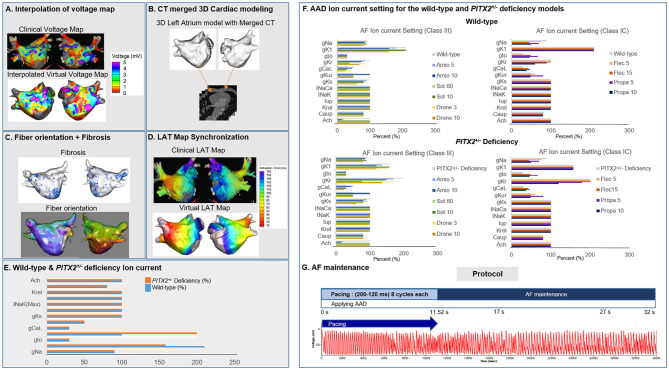
Study method for the 3D left atrial modeling. The voltage map **(A)**, CT images **(B)**, fiber orientation **(C)**, fibrosis **(C)**, and LAT synchronization **(D)** were used for the LA modeling. The wild-type and *PITX2*^+/−^ deficiency ion currents **(E,F)** was implemented to simulate for wave dynamics analysis. AF pacing protocol **(G)** was conducted to analyze the AF initiation and maintenance.

To reflect tissue characteristics in the atrial model, we performed electroanatomical modeling, fibrosis and fiber orientation modeling, and simulation setup. Electroanatomical modeling involved combining personalized CT images with voltage data. Electroanatomical maps combining clinical voltage data with CT images were used to obtain personalized 3D LA models for each study subject. We collected bipolar electrogram data for over 500 points on the left atrial (LA) surface to produce interpolated voltage data for 25 AF catheter ablation procedures (Figures 1A,B). Interpolation of clinical voltage data was used to create the virtual voltage data (Figure 1A). Such interpolation was based on the inverse distance weighting method (IDW) (Ugarte et al., 2015). The voltage data were depicted in amplitude maps obtained from bipolar electrograms with over 500 points on the left atrial (LA) surface to produce voltage. The bipolar electrograms consisted of sequential recordings of clinical electrogram during a paced rhythm with a cycle length of 500 ms. Virtual voltage data were also included in amplitude maps, which were produced by the interpolation of clinical voltage data. Interpolation of the voltage data was performed within a 10-mm radius from the region of interest. IDW was a signal interpolation method used to determine unknown data values which were weighted average of the neighboring values. IDW assumed that the closest neighboring values have the largest weight. The equation for IDW (Ugarte et al., 2015) was indicated:


(1) $$\begin{array}{l} W_{ij}=\frac{{d_{ij}}^{-a}}{\sum_{k}^{n_{j}}{d_{k}}_{j}},R_{j}=\overset{n_{j}}{\underset{i=\;1}{\sum }}w_{ij}R_{ij} \end{array}$$

where *W* represents the weighted average of neighboring values; *i* and *j* indicate the known and unknown values of points; *d*_*ij*_^−*a*^ is the distance between known and unknown points; *R*_*ij*_ represents the value of known point; and *R*_*j*_ indicates the interpolation value at unknown point *j*. Next, the voltage data were combined with CT images to create a 3D LA model map using the Ensite NavX system (Abbott Inc., Lake Bluff, IL USA). This 3D LA model map was then matched with coordinates of personalized clinical maps for accurate representation of voltage and CT images through transition and rotation. Positioning of the electroanatomical maps containing clinical voltage data and 3D LA maps onto the CT-based mesh models (Lim et al., 2020a) was conducted over four steps: geometry, trimming, field scaling, and alignment. The geometry step reflected the production of electroanatomical maps using a catheter. After the geometry step, unnecessary artifacts were removed, and the ostial position was used for separation of the LA appendage and the pulmonary vein (PV) regions during the trimming step. The field scaling step indicated the optimal scaling of interelectrode spacing and CT images. Lastly, the alignment step involved the registration of alignment points through coordinate transformation using an accurately defined ostium, along with the integration of CT images and anatomical maps. Fiber orientation environment was created using atlas-based mesh method (Ho et al., 2002) in two consecutive steps: fiber tracking and visualization. Parallel tasking was used for fiber tracking step, and visual display of fiber orientation onto 3D-LA map was conducted during visualization step. Fiber tracking was handled as an independent parallel task, which was used to determine the direction of conduction. A vector following the myocardial fiber direction could be produced at each location in the heart. The conductivity was smaller in the direction perpendicular to the vector than the conductivity in the vector direction. Fibrosis area was determined using relationship between probability of fibrosis and bipolar voltage (Zahid et al., 2016; Hwang et al., 2019) (Figure 1C). To achieve personalized virtual LA model, synchronization of clinical local activation time (LAT) map and virtual LAT map was performed. Diffusion coefficient for virtual LAT map was adjusted to accurately match conduction velocity (CV) of clinical LAT map (Lim et al., 2020a). Color scales of clinical and virtual LAT maps were compared for synchronization (Figure 1D). Finally, ion currents for sinus rhythm and AF state were set up for analysis (Figures 1E,F). The detailed protocol of AF mechanism was indicated in Figure 1G and Supplementary Materials.

### *PITX2*^+/–^ Deficiency Incorporation

The Courtemanche model (Courtemanche et al., 1998) reflecting the human atrial myocyte model was implemented for the wild-type sinus rhythm (SR) status while AF status was defined as ion current remodeling of the Courtemanche model (Lee et al., 2016). For the wild-type AF state, *I*_Na_, *I*_to_, *I*_CaL_, *I*_Kur_, and *I*_Caup_ were decreased by 10, 70, 70, 50, and 20%, and *I*_K1_ was increased by 110% compared with the Courtemanche model (Lee et al., 2016). The Syeda et al. (2016) model was used for the *PITX2*^+/−^ SR status, and the *PITX2*^+/−^ deficiency AF state was modulated with the same proportion as the wild-type SR to wild-type AF. For the *PITX2*^+/−^ deficiency AF state, *I*_Na_, *I*_to_, *I*_CaL_, *I*_Kur_, and *I*_Caup_ were decreased by 10, 70, 70, 50, and 20% whereas *I*_K1_ and *I*_Kr_ were increased by 58 and 100% compared with the Courtemanche model. The same percent change from sinus rhythm to AF for CRN was applied to normal sinus rhythm to AF for *PITX2*^+/−^ deficiency (Table 1). For the *PITX2*^+/−^ deficiency AF state, *I*_Na_, *I*_to_, *I*_CaL_, *I*_Kur_, and *I*_Caup_ were decreased by 10, 70, 70, 50, and 20%, whereas *I*_K1_ and *I*_Kr_ increased by 58 and 100%, compared with the Courtemanche model.

**Table 1:** Ion currents change for genotypes.

	**CRN**			***PITX*2** ^**+/−**^ **deficiency**		
	**Sinus rhythm** **(%)**	**AF** **(%)**	**Percent change** **(%)**	**Sinus rhythm** **(%)**	**AF** **(%)**	**Percent change** **(%)**
gNa	111	90	−19	111	90	−19
gK1	95	210	+121	71	158	+121
gto	117	30	−74	117	30	−74
gKr	120	100	−17	240	200	−17
gCaL	150	30	−80	150	30	−80
gKur	100	50	−50	100	50	−50
gKs	160	100	−38	160	100	−38
INaCa (Max)	155	100	−35	155	100	−35
INaK (Max)	100	100	0	100	100	0
Iup (Max)	100	100	0	100	100	0
Krel	100	100	0	100	100	0
Caup (Max)	125	80	−36	125	80	−36
ACh	100	100	0	100	100	0

*CRN, Courtemanche Ramirez Nattel atrial model*.

### Virtual AAD Intervention

AADs were applied to wild-type and *PITX2*^+/−^ deficiency backgrounds. Sinus rhythm and AF ion current, class III, and class IC characteristics were defined by blocking potassium and sodium channels and were compared with baseline values (Supplementary Figure 1). Ion currents for each AAD in wild-type and *PITX2*^+/−^ deficiency models are described in Table 2, and Supplementary Table 1. Supplementary Table 1 indicated the complete lists of ion currents for baselines and AADs. CRN sinus rhythm was set to 100%. Ion currents for AADs and AF status were modulated based on CRN sinus rhythm. The decrease in percentage of potassium channels and sodium channels each resembled the characteristics of class III and class IC. The references for each AAD ion current setting was described in Table 2. The degree of blocking varied within each AAD to resemble low and high dosage. The APD_90_ and CV were measured using the SR ion currents while the mean Smax, DF, PS, and AFCL were calculated using AF ion currents.

**Table 2:** References for atrial cell ion currents depending on AADs.

**AADs**	**References**	**Animal/human model**	**Method**	**Ion current change**
Amiodarone(5 μM, 10 μM)	Varela et al. (2016)	Canine atrial model	Microelectrode recording and patch-clamp	gK1, gKur, gNa, gKr, gCaL, gKs,Ach
Sotalol(60 μM, 10 mM)	Ducroq et al. (2007) Lin et al. (2007)	Rabbit/human embryonic kidney cells Xenopus oocytes	Bipolar Ag electrode recoding and patch clamp 2-electrode voltage clamp	gNa, gKr, gKs
Dronedarone(3 μM, 10 μM)	Chen et al. (2016) Gautier et al. (2003) Ji et al. (2017) Wegener et al. (2006)	Rat Guinea pig ventricular cardiomyocyte Dog ventricular myocytes Guinea pig myocytes	Whole cell, perforated patch voltage clamp	gCaL, gKs, gNa, gK1, gKr, gCaL
Flecainide(5 μM, 15 μM)	Geng et al. (2018) Yue et al. (2000) Wang et al. (1993) Hilliard et al. (2010)^11^	Human pluripotent stem cell-derived ventricular cardiomyocyte Human right atrial appendage Human pluripotent stem cell-derived ventricular cardiomyocyte Canine, murine ventricular model	Whole-cell patch voltage clamp, microscope, and confocal laser-scanning unit	gNa, gKur, gNa, gto, gCaL
Propafenone(5 μM, 10 μM)	Edrich et al. (2005) Paul et al. (2002) Seki et al. (1999) Delgado et al. (1993)	Human embryonic kidney cells Human atrial myocytes Guinea pig ventricular myocytes	Whole-cell patch voltage clamp	gNa, gto, gCaL, gKur, gKr

### Smax Evaluation, AF Induction, and Dominant Frequency Analyses

**Figure 2:**
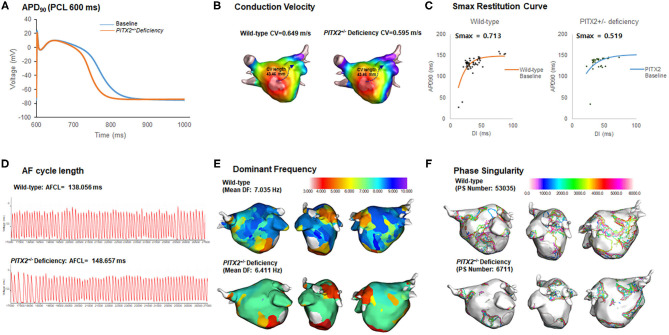
Wild-type vs. *PITX2*^+/−^ baseline model analysis. **(A–F)** The baseline APD_90_, CV, Smax, AF cycle length, and wave-dynamics parameters for wild-type and *PITX2*^+/−^ deficiency were measured for a comparison. The *PITX2*^+/−^ deficiency had a shorter APD_90_ **(A)**, lower mean Smax **(C)**, and PS number **(F)** than the wild-type.

Our graphic user interface (GUI)-based customized software (CUVIA ver. 2.5, Model: SH01; Laonmed Inc., Seoul, Korea) was utilized to visualize and analyze the action potential duration at 90% repolarization (APD_90_), conduction velocity (CV), maximal slope of the restitution curves (Smax), AF cycle length, and wave-dynamic characteristics such as the dominant frequency (DF) and phase singularity (PS). We estimated the location of the highest Smax region by generating the 3D-Smax maps. The highest Smax location matches to the high wave break points during fibrillation (Pak et al., 2003). Although precise defining of the highest Smax location was challenging, considering the heterogeneity of tissue characteristics, we localized it based on the digital numbers of Smax values in each node. A pacing cycle length of 600 ms was used to measure the APD_90_ (Song et al., 2019) and CV. The region of interest for the APD_90_ and CV was from the LA high septum (pacing sites) to the LA appendage (Figures 2A,B). Action potential duration (90%) was measured in the single cell environment. However, at the tissue level, the values of APD_90_ were heterogeneous among patients due to electroanatomical characteristics and tissue curvature of the left atrial (LA) (Song et al., 2019). The relationship between the APD_90_ and diastolic interval was plotted, and the Smax was calculated after non-linear fitting (Figure 2C) (Franz, 2003; Shattock et al., 2017). Non-linear fitting involved an exponential equation comprising a free-fitting variable, diastolic interval, action potential duration, and time constant: $y\left(Action\;potential\;duration\right)=y_{0}+A_{1}\left(1{-}^{\frac{-diastolic\;interval}{\tau_{1}}}\right)$. Details of study protocol and quantification methods for basic electrophysiologic parameters and wave-dynamic parameters, such as AF cycle length (AFCL), dominant frequency (DF), or phase singularity (PS), are available in the Supplementary Material (Figures 2D–F).

### Statistical Analyses

Categorical variables are reported as numbers (percentages), and continuous variables represent the mean with the standard deviation. We compared the changes in those parameters after virtual AADs between the two models using the Student's *t*-test. We compared the changes in the wave-dynamic parameters between the class IC and class III AADs in the overall, wild-type, and *PITX2*^+/−^ deficiency models using the Student's *t*-test. To investigate the dose-dependent effect of AADs in each model, we used the paired *t*-test to compare the change in the wave-dynamic parameters before and after the AADs with different doses. The effect sizes were calculated using Cohen's *d* (Cohen, 1988). The effect sizes were included along with *p*-values. Patients without appropriate AF wave-dynamic parameters due to termination were excluded from the statistical analysis.

## Results

### Characteristics of *PITX2*^+/–^ Deficiency AF Model

The number of cases on the tables was calculated by multiplying the number of patients and AADs with the dosage. The classes were composed of 100 cases for class IC and 150 cases for class III, and the genotype models consisted of 250 cases for each model (wild type and *PITX2*^+/−^ deficiency). Table 3 compares the electrophysiological parameters between the wide-type AF model and *PITX2*^+/−^ deficiency model, which reflected the left atrial anatomy and electrophysiology of 25 patients (68.0% male, 59.8 ± 9.8 years old, 32.0% paroxysmal AF). Study group was composed of inclusively Korean population. One patient refused genetic analysis, and two patients did not try AAD because of significant bradyarrhythmia. We did not use AADs in two patients because of associated sinus node dysfunction. AAD may aggravate their bradyarrhythmia based on the clinical decision. However, we conducted AF ablation procedures acquiring clinical electroanatomical maps. Therefore, we did not have any problem in conducting simulation studies in those two patients. The total number of patients in the study was 25. Simulation episodes included 25 cases for baseline and 250 cases with AADs.

**Table 3:** Effects of AADs in the wild-type and *PITX2*^+/−^ deficiency groups.

	**Baseline**				**Changes after AADs** **(class IC and class III)**				**Class IC**				**Class III**			
	**Wild-type (*n* = 25)**	***PITX*2**^**+/−**^ **deficiency(*n* = 25)**	***p*-value**	**Effect size**	**Wild type (*n* = 250)**	***PITX*2**^**+/−**^ **deficiency(*n*** ***= 250)***	***p*-value**	**Effectsize**	**Wild type (*n* = 100)**	***PITX*2**^**+/−**^ **deficiency(*n*** ***= 100)***	***p*-value**	**Effect size**	**Wild type (*n* = 150)**	***PITX*2**^**+/−**^ **deficiency(*n*** **= 150)**	***p*-value**	**Effect size**
APD_90_ (ms)	243.7 ± 33.8	184.4 ± 15.5	<0.001	2.553	38.2 ± 37.3	43.4 ± 56.2	0.223	0.109	275.9 ± 43.5	219.0 ± 39.2	<0.001	1.374	284.9 ± 32.8	233.8 ± 71.4	<0.001	0.919
CV, (m/s)	0.78 ± 0.32	0.70 ± 0.21	0.347	0.283	−0.15 ± 0.18	−0.20 ± 0.26	0.033	0.202	0.63 ± 0.32	0.53 ± 0.30	0.027	0.326	0.60 ± 0.36	0.43 ± 0.33	<0.001	0.513
Mean Smax	0.787 ± 0.28	0.531 ± 0.18	<0.001	1.080	0.005 ± 0.26	0.115 ± 0.24	<0.001	0.439	0.828 ± 0.31	0.694 ± 0.32	0.003	0.424	0.768 ± 0.32	0.608 ± 0.27	<0.001	0.539
Mean AFCL (ms)	146.96 ± 24.61	164.78 ± 22.73	0.011	0.752	22.62 ± 24.55	37.92 ± 32.72	<0.001	0.529	165.44 ± 36.96	190.85 ± 35.61	<0.001	0.664	169.05 ± 25.26	203.35 ± 34.78	<0.001	1.128
Peak DF (Hz)	10.68 ± 2.97	11.82 ± 3.34	0.211	0.358	−2.98 ± 4.94	−5.46 ± 4.66	<0.001	0.517	10.01 ± 4.39	7.23 ± 4.20	<0.001	0.646	6.30 ± 4.32	5.80 ± 4.07	0.301	0.120
Mean DF (Hz)	6.80 ± 0.88	6.22 ± 0.71	0.012	0.737	−1.95 ± 2.44	−2.20 ± 1.99	0.206	0.113	5.75 ± 1.78	4.53 ± 2.00	<0.001	0.645	4.14 ± 2.39	3.69 ± 2.00	0.077	0.205
PS number (*N*)	101,086 ± 96,088	14,150 ± 24,778	<0.001	1.239	−59,322 ± 99,288	−7,409 ± 27,856	<0.001	0.712	50,579 ± 65,236	11,568 ± 21,868	<0.001	0.802	32,951 ± 55,864	3,524 ± 8,302	<0.001	0.737
PS life span (ms)	109.36 ± 113.90	102.24 ± 226.64	0.889	0.040	−24.87 ± 72.06	−41.38 ± 126.35	0.073	0.161	103.36 ± 180.68	68.05 ± 162.79	0.148	0.205	71.91 ± 141.86	55.99 ± 217.97	0.454	0.087

APD_90_, action potential duration 90%; CV, conduction velocity; Smax, the maximal slope of the restitution curves; AFCL, AF cycle length; DF, dominant frequency; PS, phase singularity; n, number of patient ^*^AAD^*^ dose.

*Patients who did not sustain proper normal sinus rhythm and AF status were excluded from the analysis*.

In Table 3, we compared electrophysiological parameters at baseline, after using class IC and class III AADs, and their changes after using any AADs (including both class IC and class III AADs) with wild type and *PITX2*^+/−^ deficiency. During the baseline conditions without AADs, the *PITX2*^+/−^ deficiency group had a shorter APD_90_ at a pacing cycle length of 600 ms (*p* < 0.001, effect size = 2.553, Figure 2A), lower mean Smax during ramp pacing (*p* < 0.001, effect size = 1.080, Figure 2C), and lower mean DF (*p* = 0.012, effect size = 0.737, Figure 2E) and PS number (*p* < 0.001, effect size = 1.239, Figure 2F) after AF induction as compared with the wild type (Table 3).

### AAD Responsiveness Based on the *PITX2*^+/–^ Genotypes

We tested five different V-AADs, and the outcomes are summarized in Table 3. Changes after AADs in Table 3 indicated the effects of any AADs (including both class IC and III AADs) compared with baseline. When we compared the effects of the AADs between the wild-type and *PITX2*^+/−^ deficiency models (Table 3), the APD_90_ changes were similar (*p* = 0.223, effect size = 0.109), but the reductions in the CV (*p* = 0.033, effect size = 0.202), peak DF (*p* < 0.001, effect size = 0.517), and PS number (*p* < 0.001, effect size = 0.712) and AFCL prolongation (*p* = 0.001, effect size = 0.529) and change of Smax (*p* < 0.001, effect size = 0.439) were more significant in the *PITX2*^+/−^ deficiency model than in the wild-type AF model. For independent analyses of class IC and class III, class IC lowered APD_90_ (*p* < 0.001, Effect size = 1.374)_,_ CV (*p* < 0.027, effect size = 0.326), mean Smax (*p* = 0.003, effect size = 0.424), peak DF (*p* < 0.001, effect size = 0.646), mean DF (*p* < 0.001, effect size = 0.645), and PS number (*p* < 0.001, effect size = 0.802) in *PITX2*^+/−^ deficiency while mean AFCL (*p* < 0.001, effect size = 0.664) was increased with class IC in *PITX2*^+/−^ deficiency. Class III decreased APD_90_ (*p* < 0.001, effect size = 0.919), CV (*p* < 0.001, effect size = 0.513), mean Smax (*p* < 0.001, effect size = 0.539), and PS number (*p* < 0.001, effect size = 0.737) but increased the mean AFCL (*p* < 0.001, Effect size = 1.128) in *PITX2*^+/−^ deficiency.

### Class IC and Class III Virtual AAD Effects

**Figure 3:**
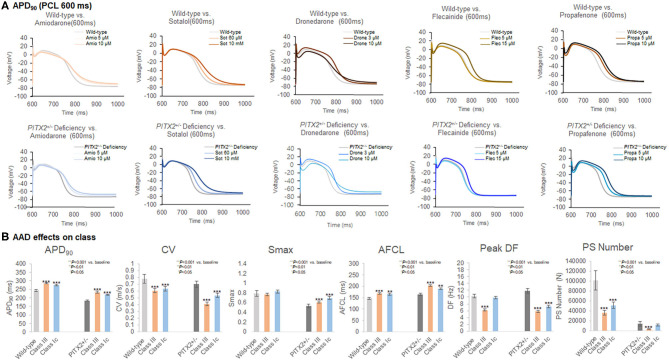
Characteristics of the wild-type and *PITX2*^+/−^ deficiency with the response to AADs. **(A,B)** The APD_90_, CV, Smax, AF cycle length, and wave-dynamics parameters were compared with baseline after AADs. **(B)** Both class III and class IC increased APD_90_, and AFCL in wild-type and *PITX2*^+−−^ deficiency model. Class III and class IC decreased CV in wild-type and *PITX2*^+/−^ deficiency model while both classes increased Smax in *PITX2*^+/−^ deficiency model.

Table 4 summarizes the effects of the virtual AADs, which included class IC AADs, flecainide, and propafenone, and the class III AADs amiodarone, sotalol, and dronedarone. At a pacing cycle length of 600 ms, the dose-dependent effect of the AADs indicated a prolonged APD_90_ with high-dose AADs (Figure 3A). The dose-dependent effects of each AAD are summarized in Supplementary Figures 2–4. APD_90_, CV, mean DF, peak DF, PS life span, PS number, Smax, and AFCL were compared for each AAD between baseline values and those after treatment. Both class III and class IC AADs changed APD_90_, CV, and AFCL, significantly compared with baseline state, regardless of wild-type or *PITX2*^+/−^ deficiency model (Figure 3B). In contrast, Smax was not changed in wild type, but rather increased in *PITX2*^+/−^ deficiency model after class III and class IC AADs (Figure 3B). The class III AADs were more effective in reducing the CV (*p* = 0.004, effect size = 0.299), the peak DF (*p* < 0.001, effect size = 0.101), mean DF (*p* < 0.001, effect size = 0.547), and PS life span (*p* = 0.020, effect size = 0.213) and prolonging of AFCL (*p* < 0.001, effect size = 0.402) than the class IC AADs (Table 4). APD_90_ prolongation (*p* = 0.040, effect size = 0.284), CV reduction (*p* = 0.003, effect size = 0.444), and AFCL lengthening (*p* = 0.002, effect size = 0.498) effects were more significant, but Smax increase was less significant (*p* = 0.010, effect size = 0.333) by class III AADs than by class IC AADs in *PITX2*^+/−^ deficiency models but not in wild-type models (Table 4).

**Table 4:** Change in the AAD effects in the wild-type and *PITX2*^+/−^ deficiency groups.

	**Overall**				**Wild type**				***PITX2***^**+/−**^ **deficiency**			
	**Class IC (*n* = 200)**	**Class III(*n* = 300)**	***p*-value**	**Effect size**	**Class IC (*n* = 100)**	**Class III(*n* = 150)**	***p*-value**	**Effect size**	**Class IC (*n* = 100)**	**Class III(*n* = 150)**	***p*-value**	**Effect size**
ΔAPD_90_ (ms)	34.1 ± 32.3	45.3 ± 55.3	0.010	0.246	33.7 ± 34.3	41.2 ± 38.9	0.124	0.202	34.5 ± 30.3	49.4 ± 67.7	0.040	0.284
ΔCV (m/s)	−0.14 ± 0.14	−0.20 ± 0.26	0.004	0.299	−0.14 ± 0.12	−0.16 ± 0.22	0.334	0.138	−0.14 ± 0.17	−0.25 ± 0.31	0.003	0.444
ΔMean Smax	0.102 ± 0.261	0.031 ± 0.250	0.003	0.277	0.041 ± 0.247	−0.020 ± 0.267	0.070	0.238	0.163 ± 0.262	0.082 ± 0.222	0.010	0.333
ΔMean AFCL (ms)	23.96 ± 28.62	35.69 ± 29.73	<0.001	0.402	19.37 ± 27.48	25.98 ± 20.72	0.075	0.271	29.40 ± 29.17	45.18 ± 33.97	0.002	0.498
ΔPeak DF (Hz)	−2.75 ± 4.85	−5.20 ± 4.80	<0.001	0.101	−0.87 ± 4.54	−4.38 ± 4.71	<0.001	0.759	−4.63 ± 4.42	−6.01 ± 4.75	0.022	0.301
ΔMean DF (Hz)	−1.38 ± 1.85	−2.53 ± 2.34	<0.001	0.547	−1.06 ± 1.80	−2.54 ± 2.64	<0.001	0.656	−1.70 ± 1.86	−2.53 ± 2.02	0.001	0.428
ΔPS number (*n*)	−25,537 ± 75,778	−38,584 ± 78,053	0.065	0.170	−48,492 ± 97,348	−66,541 ± 100,233	0.160	0.183	−2,583 ± 31,647	−10,626 ± 24,600	0.025	0.284
ΔPS life span (ms)	−20.04 ± 101.29	−41.85 ± 103.51	0.020	0.213	−6.00 ± 86.21	−37.45 ± 57.81	0.001	0.429	−34.07 ± 113.11	−46.25 ± 134.61	0.456	0.098

APD_90_, action potential duration 90%; CV, conduction velocity; Smax, the maximal slope of the restitution curves; AFCL, AF cycle length; DF, dominant frequency; PS, phase singularity; Defragmentation, termination + AT conversion; n, number of patient ^*^AAD^*^dose.

*Patients who did not maintain an AF status were excluded from the statistical analysis for the APD_90_, CV, mean Smax, mean AFCL, and wave dynamics*.

### AF Termination Under Virtual AADs

**Figure 4:**
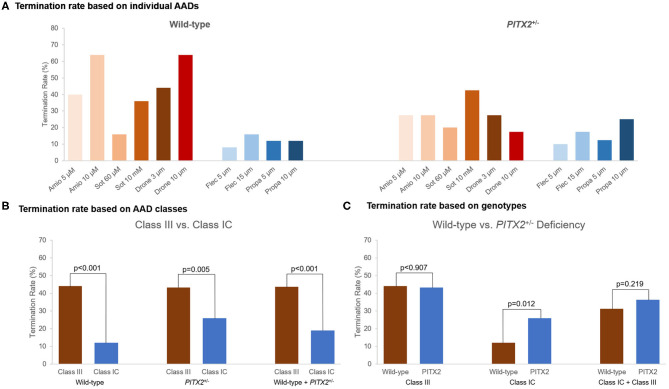
Termination rate of the wild-type and *PITX2*^+/−^ deficiency based on the AADs. **(A)** Termination rate for different AADs was described. **(B,C)** Termination rate was compared between the wild-type and *PITX2*^+/−^ deficiency and class III and class IC. **(B)** Class III showed higher AF termination rate compared with class IC. **(C)** Class IC AADs in *PITX2*^+/−^ deficiency indicated the higher AF termination rate compared with the wild type. Class III showed the higher termination rate than class IC.

AF termination was determined between 0 and 32 s after AF induction. Individual-level termination rates of AADs are described in Figure 4A. AF termination rate was significantly higher under class III AADs (43.7%) than class IC AADs (19.0%, *p* < 0.001, Figure 4B). The class IC AADs in the *PITX2*^+/−^ deficiency indicated a higher termination rate than the wild type (12.0 vs. 26.0%, *p* = 0.012, Figure 4C). Overall, the AF termination rate was 36.0% after using virtual AADs. When we compared the overall AF termination rate, there was no statistical difference between the wild-type AF (34.4%) and *PITX2*^+/−^ deficiency AF (37.6%, *p* = 0.514, Table 5). However, the *PITX2*^+/−^ deficiency AF had a statistically higher termination response to the class IC AADs (26.0%) than the wild-type AF (12.0%, *p* = 0.018, Table 5).

**Table 5:** Termination results based on the wild-type and *PITX2*^+/−^ deficiency.

	**Termination**			
	**Overall (*n* = 500)**	**Wild type(*n* = 250)**	***PITX*2**^**+/−**^ **deficiency (*n*** **= 250)**	***p*-value**
**Class IC** **+** **Class III (*****n*** **=** **500)**	180/500 (36.0%)	86/250 (34.4%)	94/250 (37.6%)	0.514
**Class IC (*****n*** **=** **200)**	38/200 (19.0%)	12/100 (12.0%)	26/100 (26.0%)	0.018
Flecainide 5 μM (*n*, %)	6 (12.0%)	2 (8.0%)	4 (16.0%)	–
Flecainide 15 μM (*n*, %)	11 (22.0%)	4 (16.0%)	7 (28.0%)	–
Propafenone 5 μM (*n*, %)	8 (16.0%)	3 (12.0%)	5 (20.0%)	–
Propafenone 10 μM (*n*, %)	13 (26.0%)	3 (12.0%)	10 (40.0%)	–
**Class III (*****n*** **=** **300)**	131/300 (43.7%)	66/150 (44.0%)	65/150 (43.3%)	1.000
Amiodarone 5 μM (*n*, %)	21 (42.0%)	10 (40.0%)	11 (44.0%)	–
Amiodarone 10 μM (*n*, %)	27 (54.0%)	16 (64.0%)	11 (44.0%)	–
Sotalol 60 μM (*n*, %)	12 (24.0%)	4 (16.0%)	8 (32.0%)	–
Sotalol 10 mM (*n*, %)	26 (52.0%)	9 (36.0%)	17 (68.0%)	–
Dronedarone 3 μM (*n*, %)	22 (44.0%)	11 (44.0%)	11 (44.0%)	–
Dronedarone 10 μM (*n*, %)	23 (46.0%)	16 (64.0%)	7 (28.0%)	–

n, number of patient ^*^AAD^*^dose.

## Discussion

### Main Findings

In this study, we invented a highly efficient patient-specific AF computation modeling system that could be applied to the virtual AAD test. We conducted a virtual AAD modeling after integrating the atrial geometry taken from the patient's cardiac CT image and electrophysiology acquired from the 3D-electroanatomical voltage map. We also evaluated the virtual AAD responsiveness by simulating the effects of five different AADs according to the *PITX2*^+/−^ genotypes. The *PITX2*^+/−^-deficient model had a shorter APD_90_, lower Smax, longer AFCL, lower mean DF, and lower PS number. The *PITX2*^+/−^ deficiency AF was easier to terminate by class IC AADs than the wild-type AF. Dose-dependent AF termination rates were significantly higher after using virtual class III AADs than class IC AADs. Although class III AADs were classified as a potassium channel blocker, amiodarone is a multichannel blocker and dronedarone is a modification of amiodarone without the iodine component. Therefore, amiodarone and dronedarone affect CV and the tissue excitability (Gautier et al., 2003; Patel et al., 2009; Saengklub et al., 2016). On the other hand, sotalol is a relatively pure potassium channel blocker (Roden, 2016), and it did not alter CV as much as amiodarone or dronedarone (Supplementary Figure 2B). The virtual AAD test was a feasible approach for evaluating the efficacy of multiple AADs in patients with AF.

### AADs in AF Rhythm Control

AADs are the most commonly used first-line therapy for AF rhythm control. Nevertheless, <50% of AF patients maintain adequate SR for more than 1 year with AADs, which is significantly lower than in AF catheter ablation (Singh et al., 2005). The first obstacle to the proper use of AADs is the risk of various adverse effects such as sinus node dysfunction, proarrhythmias, and an increased mortality, and the second is the difference in the drug effects, depending on the patient characteristics (Parvez et al., 2012). Because of this, a sufficient dose of the AAD carries the potential risk of side effects, while an excessively careful reduced-dose AAD administration lowers the effect of the rhythm control. The virtual AAD simulation test considered the personal and genetic characteristics of the AF patients, and therefore, it has the potential as an important breakthrough for more efficient AF drug therapy in the future.

### *PITX2* Variant Characteristics

AF is already well-known as a heritable disease (Lubitz et al., 2010). In particular, the *PITX2* gene has been known to be the universal first AF-associated SNP in European, Japanese, and Korean populations (Lee et al., 2017; Low et al., 2017; Roselli et al., 2018). Unique electrophysiological changes that create AF vulnerable conditions are observed in *PITX2*^+/−^-deficient animal models. First, a *PITX2*^+/−^ deficiency results in a condition with a shortening of the APD by reducing the I_K1_ and I_Ca−L_ and by increasing the I_Ks_ (Syeda et al., 2016). Second, it generates a slower CV by raising the atrial cell resting membrane potential and changing the gap junctional conduction (Chinchilla et al., 2011). Third, the *PITX2*^+/−^ deficiency is related to triggered activity caused by abnormal calcium management (Denham et al., 2018).

Parvez et al. (2012) reported that rs10033464 in the *PITX2* gene is independently associated with the response to the AAD treatment in patients with AF. This report is in agreement with a study by Syeda et al. (2016) on the sensitivity to flecainide, a sodium channel blocker, in the *PITX2* deficiency animal model (Parvez et al., 2012). In this study, we observed significant electrophysiological changes related to the *PITX2* deficiency and its responsiveness to class IC AADs in the AF computational modeling of 25 patients, which was consistent with the previous studies (Syeda et al., 2016). Moreover, the AF wave dynamics, including the Smax also exhibited characteristic changes according to the genetic traits or specific AADs.

### Virtual AAD Modeling in AF

Since the first human AF computational modeling was presented by Moe et al. (1964), sophisticated simulation modeling that integrates the patient's anatomical, histological, and electrophysiological characteristics have been utilized in various physiological studies (Trayanova, 2014). The biggest obstacle to the clinical use of a sophisticated AF computational modeling so far has been the long computational speed of a complex simulation. However, recent innovations in the hardware and software have opened the gate for the clinical utilization of AF computational modeling (Kwon et al., 2013). Lim et al. (2020b) used a graphic process unit to analyze the AF modeling and a wave-dynamics analysis within 45 min while considering the patient's anatomy (cardiac computed tomogram), electrophysiology (3D-electroanatomical map), fibrosis (voltage map), and fiber orientation (LAT map). In this study, we further clinically applied the computational modeling by testing five different virtual AADs, depending on the *PITX2* genotypes, in a realistic AF modeling in 25 patients.

### Limitations

The LA model used a personalized/realistic electroanatomy, fibrosis, and fiber orientation; however, the LA model was designed to be a monolayer. Implementation of epicardial conduction could provide the endocardial-acquired local activation pattern and clinical voltage. Including the atrial wall thickness in the LA model could provide more accurate representation of the wave-dynamics analyses, thus, the results would be a more clinically applicable LA model. Bilayers, including both endocardial and epicardial layers, were not reflected in the model (Labarthe et al., 2014). The fiber orientation can be a simplistic version of a rather sophisticated image or a rule-based approach of the fiber orientation (Krueger et al., 2013). Multisite induction could be conducted to reflect the complicated AF dynamics (Prakosa et al., 2018). The majority of the references for the *PITX2*^+/−^ deficiency and AAD-induced changes of the trans-membrane ion currents were based on *in vivo* experiments with animal models. In this study, a detailed analysis of sarcoplasmic reticular calcium leaking and triggered activity was not performed. There are still obstacles to this approach, such as the need for invasive mapping data. Invasive LA modeling was conducted, whereas other modeling using LDGE MRI (Lopez-Perez et al., 2015) or ECGi (Perez Alday et al., 2019) could be considered for further study.

### Conclusion

We conducted a virtual AAD test with five different AADs, according to the *PITX2*^+/−^ genotype, after integrating the atrial geometry taken from the patient's cardiac CT image and electrophysiology acquired from a 3D-electroanatomical voltage map. The *PITX2*^+/−^-deficient model exhibited different electrophysiology and AF wave dynamics than the wild type. *PITX2*^+/−^ deficiency AF was easier to terminate by class IC AADs than the wild-type AF. Therefore, the virtual AAD test was a feasible approach for evaluating the efficacy of multiple AADs in patients with AF.

## Data Availability Statement

The raw data supporting the conclusions of this article will be made available by the authors, without undue reservation.

## Ethics Statement

The studies involving human participants were reviewed and approved by the institutional Review Board of the Yonsei University Health System. The patients/participants provided their written informed consent to participate in this study.

## Author Contributions

H-NP participated in the study design, study protocol design, data review, and writing manuscript. IH and ZJ was involved in the data collection, analysis, and writing manuscript. J-WP was involved in the data interpretation and statistical analysis. O-SK participated in the study tool design. BL was involved in the data collection, and study protocol design. MH, MK, and H-TY participated in the data interpretation. T-HK, J-SU, BJ, and M-HL were involved in the data interpretation and data review. All authors contributed to the article and approved the submitted version.

## Conflict of Interest

The authors declare that the research was conducted in the absence of any commercial or financial relationships that could be construed as a potential conflict of interest.
