# Corrigendum: Computational modeling for antiarrhythmic drugs for atrial fibrillation according to genotype

**DOI:** 10.3389/fphys.2022.991997

**Published:** 2022-12-02

**Authors:** Inseok Hwang, Ze Jin, Je-Wook Park, Oh-Seok Kwon, Byounghyun Lim, Myunghee Hong, Min Kim, Hee-Tae Yu, Tae-Hoon Kim, Jae-Sun Uhm, Boyoung Joung, Moon-Hyoung Lee, Hui-Nam Pak

**Affiliations:** Yonsei University Health System, Seoul, South Korea

**Keywords:** atrial fibrillation, modeling, antiarrhythmic drugs, *PITX2*, gene

In the published article, there was an error in the **Author List**. Author “Ze Jin” was erroneously excluded. The corrected author list appears below:

“Inseok Hwang; Ze Jin; Je-Wook Park; Oh-Seok Kwon; Byounghyun Lim; Myunghee Hong; Min Kim; Hee-Tae Yu; Tae-Hoon Kim; Jae-Sun Uhm; Boyoung Joung; Moon-Hyoung Lee; Hui-Nam Pak.”

In the published article, there was an error in Supplementary Table S1. The ion currents for baselines and AADs were described incorrectly. The corrected Supplementary Table S1 appears in the Supplementary material.

In the published article, there was an error. The **Author Contributions** were described incorrectly:

“H-NP was participated in the study design, study protocol design, data review, and writing manuscript. IH was involved in the data collection, analysis, and writing manuscript. J-WP was involved in the data interpretation and statistical analysis. O-SK was participated in the study tool design. BL was involved in the data collection, and study protocol design. MH, MK, and H-TY were participated in the data interpretation. T-HK, J-SU, BJ, and M-HL were involved in the data interpretation and data review. All authors contributed to the article and approved the submitted version.”

The corrected **Author Contributions** statement appears below:

“H-NP participated in the study design, study protocol design, data review, and writing manuscript. IH and ZJ was involved in the data collection, analysis, and writing manuscript. J-WP was involved in the data interpretation and statistical analysis. O-SK participated in the study tool design. BL was involved in the data collection, and study protocol design. MH, MK, and H-TY participated in the data interpretation. T-HK, J-SU, BJ, and M-HL were involved in the data interpretation and data review. All authors contributed to the article and approved the submitted version.”

The authors apologize for these errors and state that this does not change the scientific conclusions of the article in any way. The original article has been updated.

