# Supplementary material for: Computational Modeling for Antiarrhythmic Drugs for Atrial Fibrillation According to Genotype
Source: Front Physiol. 2021 May 13;12:650449. doi: 10.3389/fphys.2021.650449 (PMC8155488; doi:10.3389/fphys.2021.650449)
Supplement: Supplementary file 1 [file Data_Sheet_1.docx]

***Supplementary Material***

**1 Methods**

**1.1 Smax evaluation, AF induction, and Dominant Frequency analyses**

To induced AF, ramp pacing from 200 ms to 120 ms for 11.52 s was conducted and the pacing site was determined based on the earliest activation site of the clinical LAT maps. After induction of AF by ramp pacing, we analyzed the AF cycle length and AF wave-dynamics parameters by sustaining AF, recorded for up to 32 s, including a 6.48 s post-pacing blanking period (Figure 1G, Figure 2D~F). In this model, the 32 s included the induction, blanking, and AF maintenance periods. The induction period was from 0-11.52 s, and the blanking period occurred during the 11.52-17 s window. The maintenance period occurred after the blanking period up until 32 s. We calculated the mean AF cycle length by the total number of cycles divided by 10 s, which was an AF recording of 17-27 s per period (Figure 2D). The time window for AF wave-dynamics such as the dominant frequency (DF) and phase singularity (PS) was set between 17-23 s. The DF analysis was divided into an evaluation of the DF with the highest frequency and average frequency of the 3D-DF maps (Figure 2E). A Hanning window was utilized to determine the Fourier transformation of the action potentials at each node and power spectra density function (Lim et al., 2017). The DF was quantitatively analyzed for each node and visually displayed on GUI based software as 3D maps. PS number and PS life span were measured using our validated location-centric method (Lee et al., 2016). Iver-Gray’s algorithm was used to calculate PS number (Iyer-Gray et al., 2001). PS number was calculated for 6 seconds. PS life span was defined as the average amount of time that the PS lasted in the atrium. PS number was the number of PS points found in the atrial region per ms (Figure 2F and Supplementary Figure 4).

**Supplementary material reference**

Iyer, A.N., and Gray, R.A. (2001). An experimentalist's approach to accurate localization of phase singularities during reentry. *Ann Biomed Eng* 29**,** 47-59.

Lee, Y.-S., Song, J.-S., Hwang, M., Lim, B., Joung, B., and Pak, H.-N. (2016). A New Efficient Method for Detecting Phase Singularity in Cardiac Fibrillation. *PLOS ONE* 11**,** e0167567.

Lim, B., Hwang, M., Song, J.-S., Ryu, A.-J., Joung, B., Shim, E.B., Ryu, H., and Pak, H.-N. (2017). Effectiveness of atrial fibrillation rotor ablation is dependent on conduction velocity: An in-silico 3-dimensional modeling study. *PLOS ONE* 12**,** e0190398.

**Supplementary Table 1. AAD ion current setting for CRN and *PITX2*^+/-^ deficiency**

|  |  | **CRN^‡^ sinus rhythm** | | | | | | | | | |
| --- | --- | --- | --- | --- | --- | --- | --- | --- | --- | --- | --- |
|  | **Baseline** | **Amiodarone**  **5 uM (%)** | **Amiodarone 10 uM (%)** | **Sotalol**  **60 uM (%)** | **Sotalol**  **10 mM (%)** | **Dronedarone 3 uM (%)** | **Dronedarone 10 uM (%)** | **Flecainide**  **5 uM (%)** | **Flecainide 15 uM (%)** | **Propafenone 5 uM (%)** | **Propafenone 10 uM (%)** |
| **gNa** | 100 | 100 | 94 | 86 | 100 | 100 | 90 | 83 | 50 | 78 | 56 |
| **gK1** | 100 | 86 | 76 | 100 | 100 | 95 | 76 | 100 | 100 | 100 | 100 |
| **gto** | 100 | 100 | 100 | 100 | 100 | 100 | 100 | 100 | 63 | 70 | 50 |
| **gKr** | 100 | 85 | 75 | 70 | 38 | 85 | 68 | 100 | 100 | 90 | 60 |
| **gCaL** | 100 | 50 | 40 | 100 | 100 | 40 | 27 | 100 | 70 | 50 | 40 |
| **gKur** | 100 | 100 | 100 | 100 | 100 | 100 | 100 | 40 | 20 | 70 | 50 |
| **gKs** | 100 | 90 | 80 | 90 | 80 | 80 | 60 | 100 | 100 | 100 | 100 |
| **INaCa (Max)** | 100 | 100 | 100 | 100 | 100 | 100 | 100 | 100 | 100 | 100 | 100 |
| **INaK (Max)** | 100 | 100 | 100 | 100 | 100 | 100 | 100 | 100 | 100 | 100 | 100 |
| **Iup (Max)** | 100 | 100 | 100 | 100 | 100 | 100 | 100 | 100 | 100 | 100 | 100 |
| **Krel** | 100 | 100 | 100 | 100 | 100 | 100 | 100 | 100 | 100 | 100 | 100 |
| **Caup (Max)** | 100 | 100 | 100 | 100 | 100 | 100 | 100 | 100 | 100 | 100 | 100 |
| **Ach** | 100 | 22 | 15 | 100 | 100 | 100 | 100 | 100 | 100 | 100 | 100 |
|  |  | **CRN^‡^ AF** | | | | | | | | | |
| **gNa** | 90 | 90 | 85 | 77 | 90 | 90 | 81 | 75 | 45 | 70 | 50 |
| **gK1** | 210 | 180 | 160 | 210 | 210 | 200 | 160 | 210 | 210 | 210 | 210 |
| **gto** | 30 | 30 | 30 | 30 | 30 | 30 | 30 | 30 | 19 | 21 | 15 |
| **gKr** | 100 | 85 | 75 | 70 | 38 | 85 | 68 | 100 | 100 | 90 | 60 |
| **gCaL** | 30 | 15 | 12 | 30 | 30 | 12 | 8 | 30 | 21 | 15 | 12 |
| **gKur** | 50 | 50 | 50 | 50 | 50 | 50 | 50 | 20 | 10 | 35 | 25 |
| **gKs** | 100 | 90 | 80 | 90 | 80 | 80 | 60 | 100 | 100 | 100 | 100 |
| **INaCa (Max)** | 100 | 100 | 100 | 100 | 100 | 100 | 100 | 100 | 100 | 100 | 100 |
| **INaK (Max)** | 100 | 100 | 100 | 100 | 100 | 100 | 100 | 100 | 100 | 100 | 100 |
| **Iup (Max)** | 100 | 100 | 100 | 100 | 100 | 100 | 100 | 100 | 100 | 100 | 100 |
| **Krel** | 100 | 100 | 100 | 100 | 100 | 100 | 100 | 100 | 100 | 100 | 100 |
| **Caup (Max)** | 80 | 80 | 80 | 80 | 80 | 80 | 80 | 80 | 80 | 80 | 80 |
| **Ach** | 100 | 22 | 15 | 100 | 100 | 100 | 100 | 100 | 100 | 100 | 100 |
|  | ***PITX2*^+/-^ Deficiency sinus rhythm** | | | | | | | | | | |
| **gNa** | 100 | 100 | 94 | 86 | 100 | 100 | 90 | 83 | 50 | 78 | 56 |
| **gK1** | 75 | 64 | 57 | 75 | 75 | 71 | 57 | 75 | 75 | 75 | 75 |
| **Gto** | 100 | 100 | 100 | 100 | 100 | 100 | 100 | 100 | 63 | 70 | 50 |
| **gKr** | 200 | 170 | 150 | 140 | 76 | 170 | 136 | 200 | 200 | 180 | 120 |
| **gCaL** | 100 | 50 | 40 | 100 | 100 | 40 | 27 | 100 | 70 | 50 | 40 |
| **gKur** | 100 | 100 | 100 | 100 | 100 | 100 | 100 | 40 | 20 | 70 | 50 |
| **gKs** | 100 | 90 | 80 | 90 | 80 | 80 | 60 | 100 | 100 | 100 | 100 |
| **INaCa (Max)** | 100 | 100 | 100 | 100 | 100 | 100 | 100 | 100 | 100 | 100 | 100 |
| **INaK (Max)** | 100 | 100 | 100 | 100 | 100 | 100 | 100 | 100 | 100 | 100 | 100 |
| **Iup (Max)** | 100 | 100 | 100 | 100 | 100 | 100 | 100 | 100 | 100 | 100 | 100 |
| **Krel** | 100 | 100 | 100 | 100 | 100 | 100 | 100 | 100 | 100 | 100 | 100 |
| **Caup (Max)** | 100 | 100 | 100 | 100 | 100 | 100 | 100 | 100 | 100 | 100 | 100 |
| **Ach** | 100 | 22 | 15 | 100 | 100 | 100 | 100 | 100 | 100 | 100 | 100 |
|  | ***PITX2*^+/-^ Deficiency AF** | | | | | | | | | | |
| **gNa** | 90 | 90 | 85 | 77 | 90 | 90 | 81 | 75 | 45 | 70 | 50 |
| **gK1** | 158 | 135 | 120 | 158 | 158 | 150 | 120 | 158 | 158 | 158 | 158 |
| **gto** | 30 | 30 | 30 | 30 | 30 | 30 | 30 | 30 | 19 | 21 | 15 |
| **gKr** | 200 | 170 | 150 | 140 | 76 | 170 | 136 | 200 | 200 | 180 | 120 |
| **gCaL** | 30 | 15 | 12 | 30 | 30 | 12 | 8 | 30 | 21 | 15 | 12 |
| **gKur** | 50 | 50 | 50 | 50 | 50 | 50 | 50 | 20 | 10 | 35 | 25 |
| **gKs** | 100 | 90 | 80 | 90 | 80 | 80 | 60 | 100 | 100 | 100 | 100 |
| **INaCa (Max)** | 100 | 100 | 100 | 100 | 100 | 100 | 100 | 100 | 100 | 100 | 100 |
| **INaK (Max)** | 100 | 100 | 100 | 100 | 100 | 100 | 100 | 100 | 100 | 100 | 100 |
| **Iup (Max)** | 100 | 100 | 100 | 100 | 100 | 100 | 100 | 100 | 100 | 100 | 100 |
| **Krel** | 100 | 100 | 100 | 100 | 100 | 100 | 100 | 100 | 100 | 100 | 100 |
| **Caup (Max)** | 80 | 80 | 80 | 80 | 80 | 80 | 80 | 80 | 80 | 80 | 80 |
| **Ach** | 100 | 22 | 15 | 100 | 100 | 100 | 100 | 100 | 100 | 100 | 100 |

**^‡^**CRN refers to Courtemanche Ramirez Nattel atrial model.

**Supplementary Figure legends**

**Supplementary Figure 1. Ion current settings for each AAD**

**(A-B)** The ion current settings for amiodarone 5 μM, amiodarone 10 μM, sotalol 60 μM, sotalol 10 mM, dronedarone 3 μM, dronedarone 10 μM, flecainide 5 μM, flecainide 15 μM, propafenone 5 μM, and propafenone 10 μM were displayed in the wild-type AF state, and *PITX2*^+/-^ deficiency AF state.

**Supplementary Figure 2. Dose dependent effect of AADs**

**(A-D)** The dose dependent effect was determined at baseline. **(A)** The APD_90_ was the highest for sotalol 10 mM in both the wild-type and *PITX2*^+/-^ deficiency group. **(B)** The CV was the slowest with amiodarone 10 μM in the wild-type and dronedarone 10 μM in *PITX2*^+/-^ deficiency group. **(C)** The mean DF was the lowest with amiodarone 10 μM in the wild-type and sotalol 10 mM in the *PITX2*^+/-^ deficiency group. **(D)** The peak DF was lowest with amiodarone 10 μM in the wild-type group and sotalol 10 mM in the *PITX2*^+/-^ deficiency group. The standard errors were used for the error bars. **(E)** PS life span was shortest with dronedarone 10 μM in wild-type and sotalol 10mM in the *PITX2*^+/-^ deficiency group. **(F)** PS number was lowest with dronedarone 10 μM in wild-type and amiodarone 5 μM in the *PITX2*^+/-^ deficiency group. **(G)** Smax was lowest in amiodarone 5 μM in wild-type and highest in propafenone 10 μM. **(H)** AFCL was the highest in amiodarone 10 μM in both wild-type and the *PITX2*^+/-^ deficiency group.

**Supplementary Figure 3. 3D DF and Smax map**

**(A-B)** The representative examples of 3D DF and Smax Map based on genotypes and AADs

**Supplementary Figure 4. 3D PS map**

**(A)** The representative examples of 3D PS Map based on genotypes and AADs
