# Supplementary figures and images for: Computational Modeling for Antiarrhythmic Drugs for Atrial Fibrillation According to Genotype
Source: Front Physiol. 2021 May 13;12:650449. doi: 10.3389/fphys.2021.650449 (PMC8155488; doi:10.3389/fphys.2021.650449)

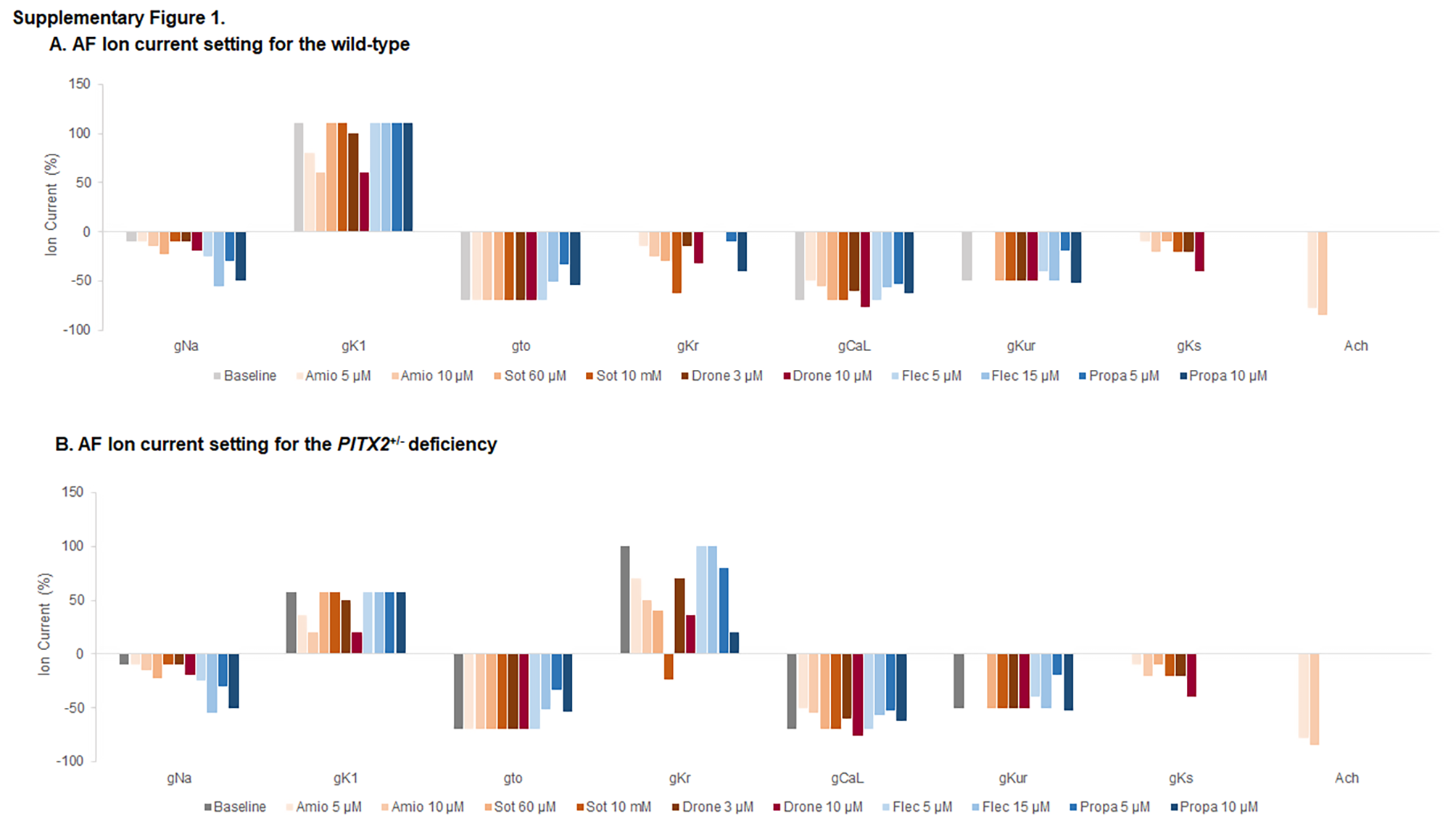

Supplement: Supplementary file 2 [file Image_1.TIF]

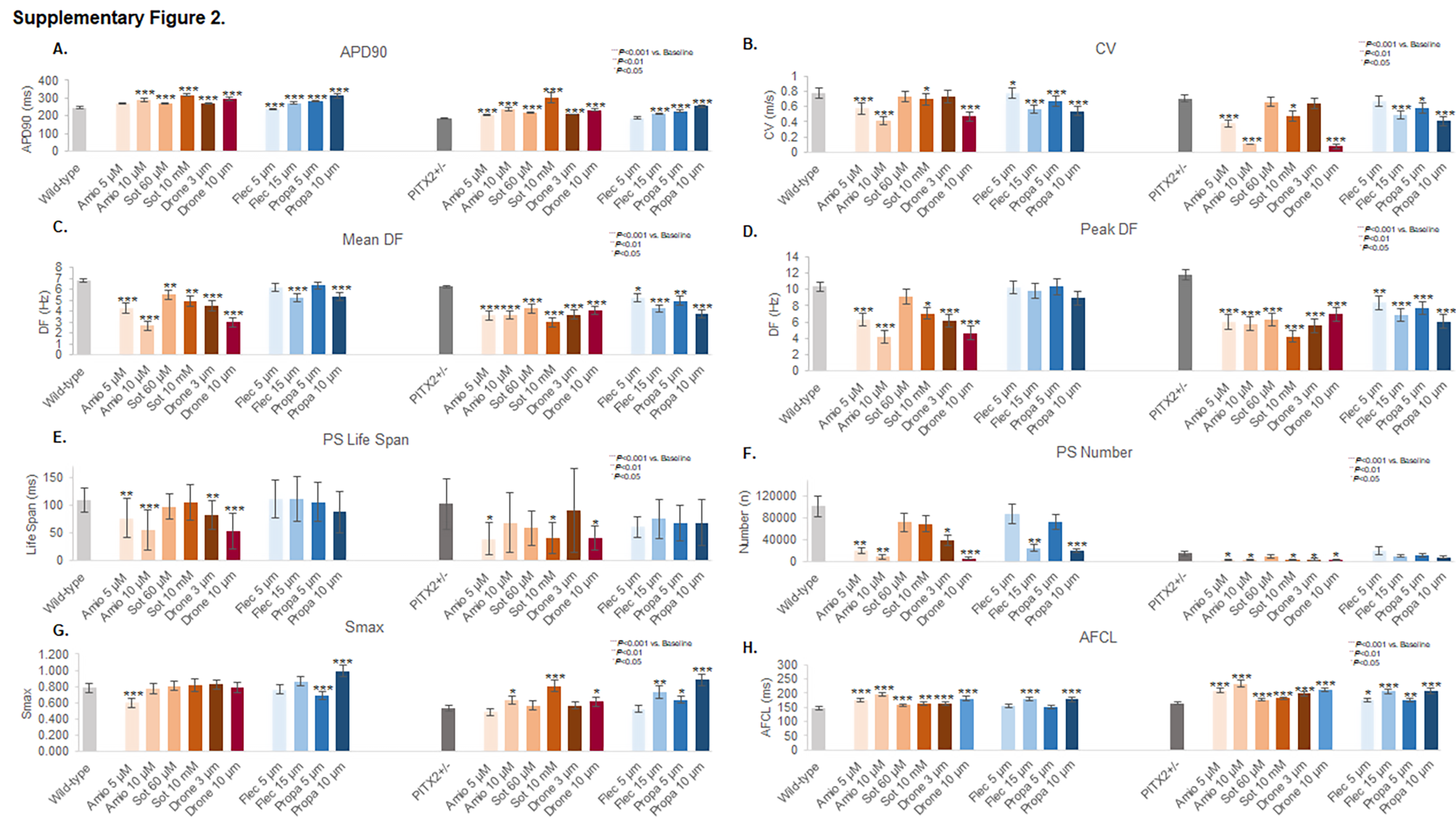

Supplement: Supplementary file 3 [file Image_2.TIF]

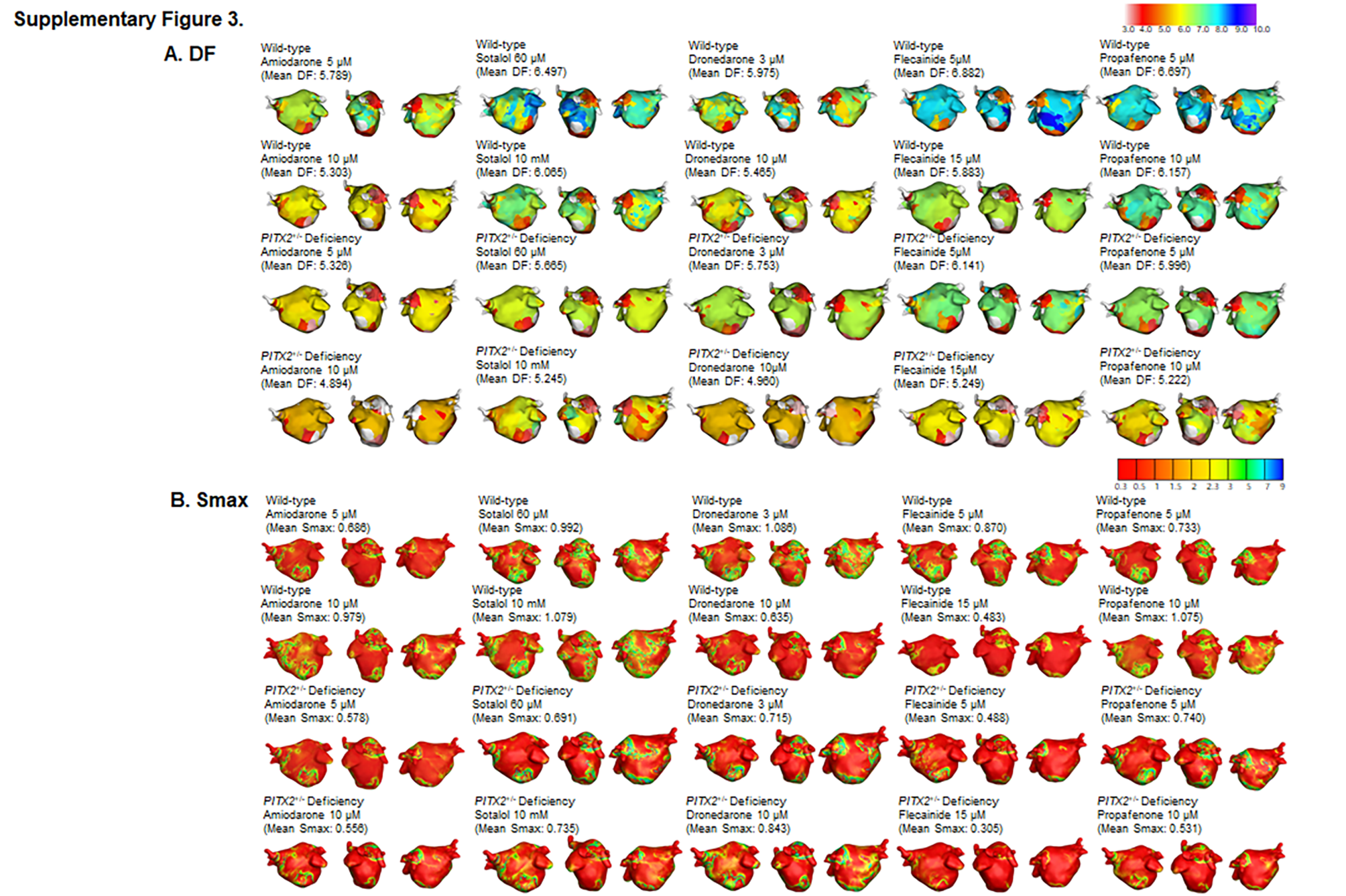

Supplement: Supplementary file 4 [file Image_3.TIF]

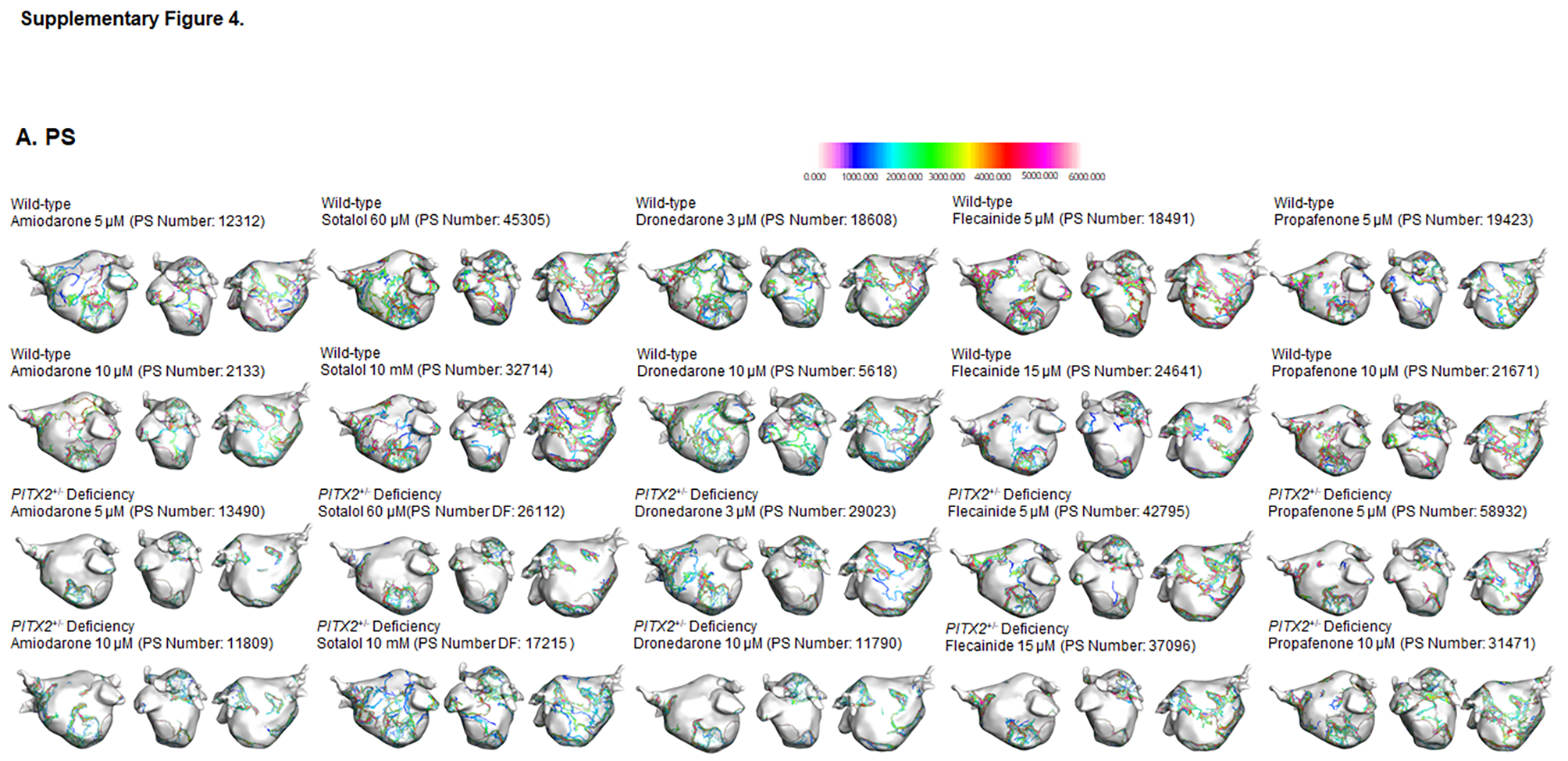

Supplement: Supplementary file 5 [file Image_4.TIF]
